# Revisiting unexploited antibiotics in search of new antibacterial drug candidates: the case of γ-actinorhodin

**DOI:** 10.1038/s41598-017-17232-1

**Published:** 2017-12-12

**Authors:** Nada M. Nass, Sannia Farooque, Charlotte Hind, Matthew E. Wand, Christopher P. Randall, J. Mark Sutton, Ryan F. Seipke, Christopher M. Rayner, Alex J. O’Neill

**Affiliations:** 1 Antimicrobial Research Centre and School of Molecular and Cellular Biology, Faculty of Biological Sciences, University of Leeds, Leeds, LS2 9JT UK; 2 School of Chemistry, University of Leeds, Leeds, LS2 9JT UK; 3 Public Health England, National Infection Service, Porton Down, Salisbury, Wiltshire SP4 0JG UK

## Abstract

Of the thousands of natural product antibiotics discovered to date, only a handful have been developed for the treatment of bacterial infection. The clinically unexploited majority likely include compounds with untapped potential as antibacterial drugs, and in view of the ever-growing unmet medical need for such agents, warrant systematic re-evaluation. Here we revisit the actinorhodins, a class that was first reported 70 years ago, but which remains poorly characterized. We show that **γ**-actinorhodin possesses many of the requisite properties of an antibacterial drug, displaying potent and selective bactericidal activity against key Gram-positive pathogens (including *Staphylococcus aureus* and enterococci), a mode of action distinct from that of other agents in clinical use, an extremely low potential for the development of resistance, and a degree of *in vivo* efficacy in an invertebrate model of infection. Our findings underscore the utility of revisiting unexploited antibiotics as a source of novel antibacterial drug candidates.

## Introduction

The growing prevalence of antibiotic resistance in pathogenic bacteria is severely eroding our ability to manage bacterial infection^1^. Central to an effective response to this problem will be the development of novel antibacterial drugs that display activity against bacteria resistant to existing antibiotics. Unfortunately, progress towards this end has been hindered by the extremely challenging nature of antibacterial drug discovery; the field is now 30 years into a ‘Discovery Void’, from which no novel drug class effective against the problematic ESKAPE pathogens (*Enterococcus faecium, Staphylococcus aureus, Klebsiella pneumoniae, Acinetobacter baumannii, Pseudomonas aeruginosa*, and *Enterobacter* species) has emerged to reach the clinic^2^.

A strategy that we have been pursuing with a view to identifying new antibacterial drug candidates involves systematically revisiting known classes of natural product antibiotics that have not to date been exploited for the treatment of bacterial infection^3^. The underpinning hypothesis is that amongst the *c*. 3000 antibiotics discovered to date^4^ - of which only a handful of classes have been deployed clinically – there may reside a wealth of compounds with untapped potential as antibacterial drugs. By initiating the discovery process with compounds about which something is already known, including the fact that they possess antibacterial activity, this approach offers a potential fast-track through the challenging early stages of discovery. Corroborating the viability of such a strategy there are several examples of clinically-useful antibiotics that were dismissed as drug candidates following their discovery, but which were later successfully developed for therapeutic use (daptomycin, fidaxomicin, pleuromutilins)^2^.

Actinorhodin (ACT), a dimeric benzoisochromanequinone antibiotic produced by *Streptomyces coelicolor* A3(2), was first reported in the late 1940’s^5^. It was subsequently shown that *S. coelicolor* produces a series of closely-related compounds in addition to ACT itself ^6^, which are collectively referred to here as the actinorhodins (ACTs) or the ACT class. Most studies on this compound appear to have employed mixtures of ACTs, since the methods routinely used to purify and characterize ACT are insufficient to resolve it from its close analogues^6–9^. ACT and other members of the class exhibit some intriguing characteristics; they are strongly pigmented and demonstrate litmus-like properties, undergoing a reversible colour change from blue under alkaline conditions to red in acid^8^, and are capable of acting as organocatalysts to drive oxidation reactions *in vitro* ^7^. ACTs lend colonies of *S. coelicolor* their characteristic blue colour, and since the presence of these compounds is therefore easily detected by eye, ACT production has been widely employed as a phenotypic marker in streptomycete research^10,11^.

Only limited information exists regarding the antibacterial properties of ACTs. Weak antibacterial activity has been reported against some Gram-positive bacteria, with an estimated MIC against *S. aureus* of 25–30 µg/ml^12^. However, the experiments from which these results derive, involving measuring zones of inhibition around transplanted plugs of agar on which *S. coelicolor* had been grown, mean that these values must be considered approximate at best. There is also no clear understanding of the antibacterial mode of action of ACTs, although more than one hypothesis has been put forward. Naturally-occurring quinones, such as ACT, can function as bioreductive DNA-alkylating agents^13,14^, a property that could potentially explain ACT’s antibacterial action. Alternatively, a recent study proposed that ACT exerts its antibacterial effect by catalysing the production of toxic levels of hydrogen peroxide^7^.

Here we sought to more comprehensively investigate the antibacterial properties of this class, with a view to evaluating the potential of the ACT pharmacophore for use in antibacterial chemotherapy. In doing so, we elected to work with a single, defined species of ACT to avoid the potential confounding associated with studying a mixture of ACT analogues that might vary considerably in respect of their antibacterial properties. This strategy therefore required that we first define a particular species of ACT for study. Proceeding on the basis that *S. coelicolor* likely produces ACT as a defensive molecule, we identified and purified the predominant secreted (extracellular) form of ACT with the aim of maximising our chances of working with the congener exhibiting the greatest antibacterial potency. The studies on this compound (**γ**-actinorhodin) that we present here reveal that the ACT pharmacophore in fact displays potent antistaphylococcal activity, and possesses many of the requisite properties of a useful antibacterial drug.

## Results

### Isolation and purification of γ-actinorhodin (γ-ACT)

**Figure 1:**
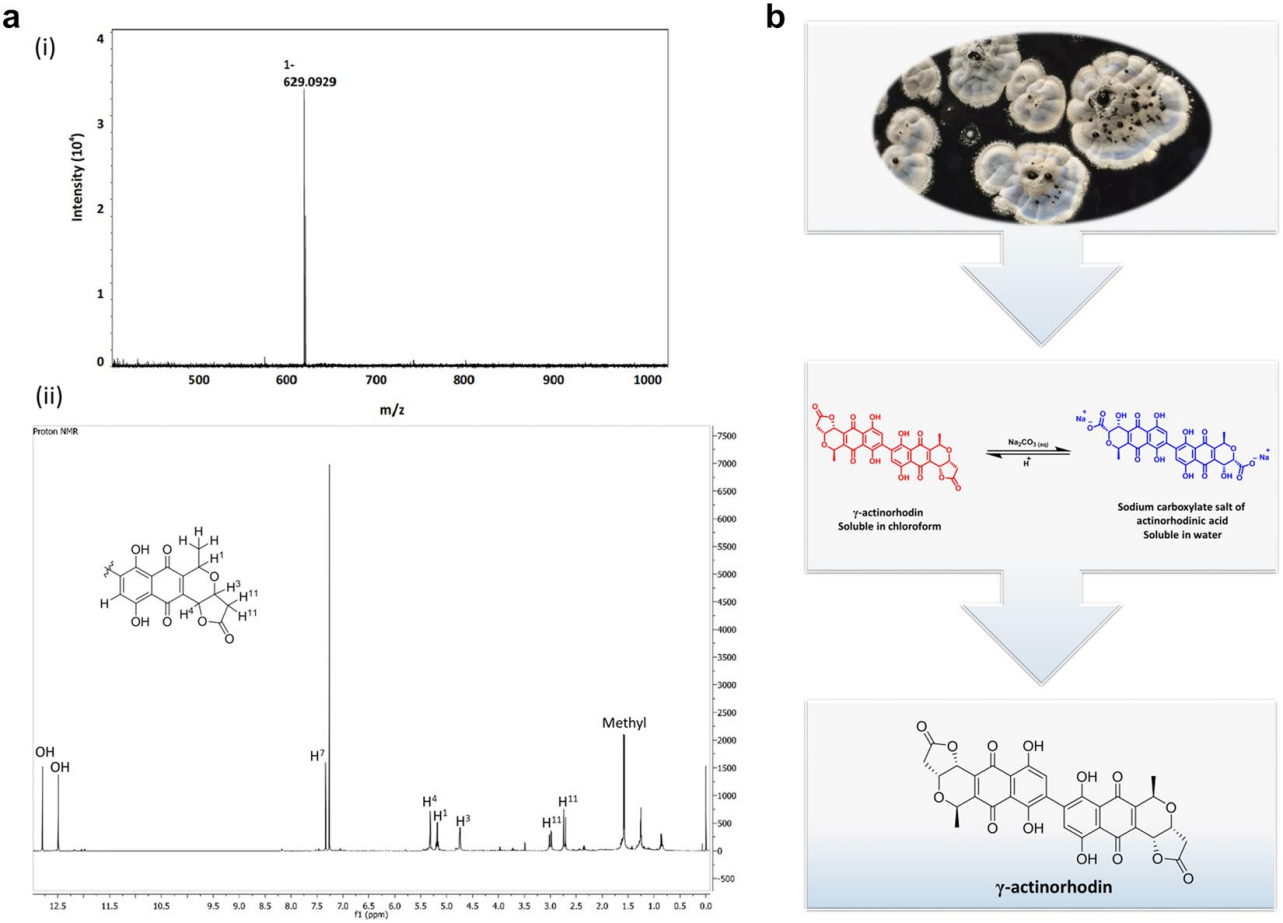
Identification of γ-ACT as the predominant ACT species secreted by *S. coelicolor* L646 using LC-MS (**ai**) and ^1^H NMR (**aii**), and overview of the γ-ACT purification strategy (**b**).

After 4–6 days’ growth on ISP 2 agar, *S. coelicolor* L646 turns the medium blue owing to ACT secretion. Preliminary TLC profiling of a crude extract of the agar revealed several pigments exhibiting the litmus-like properties of ACTs; the dominant, well-separated component at R_f_ 0.29 in dichloromethane-methanol 9:1 (v/v) was collected and characterized by ^1^H NMR spectroscopy and LC-MS, which identified it as γ-ACT (Fig. 1a). Subsequently, a scalable method was devised for the targeted isolation and purification of this compound (Fig. 1b). On the basis that the lactone substituent of γ-ACT makes this molecule substantially less polar compared with other ACTs, γ-ACT was extracted directly from agar using ethyl acetate, leaving behind the more polar congeners; ^1^H NMR analysis of the crude ethyl acetate extract confirmed the presence of γ-ACT alongside unknown impurities. We anticipated that at high pH (>10), γ-ACT would selectively ring-open to afford the hydroxycarboxylate (the salt of actinorhodinic acid), a compound with high water solubility. Washing the crude extract with aqueous sodium carbonate (pH 10.9) resulted in a blue aqueous layer, which was separated and then acidified with hydrochloric acid (6 M) to reform and precipitate the lactone. γ-ACT was triturated from methanol as a red solid in 19% yield. The identity and purity (>95%) of γ-ACT were confirmed using mass spectrometry, 1-D NMR, 2-D NMR, infrared spectroscopy and analytical HPLC (*supplementary information*).

### *In vitro* antibacterial activity of γ-ACT

**Figure 2:**
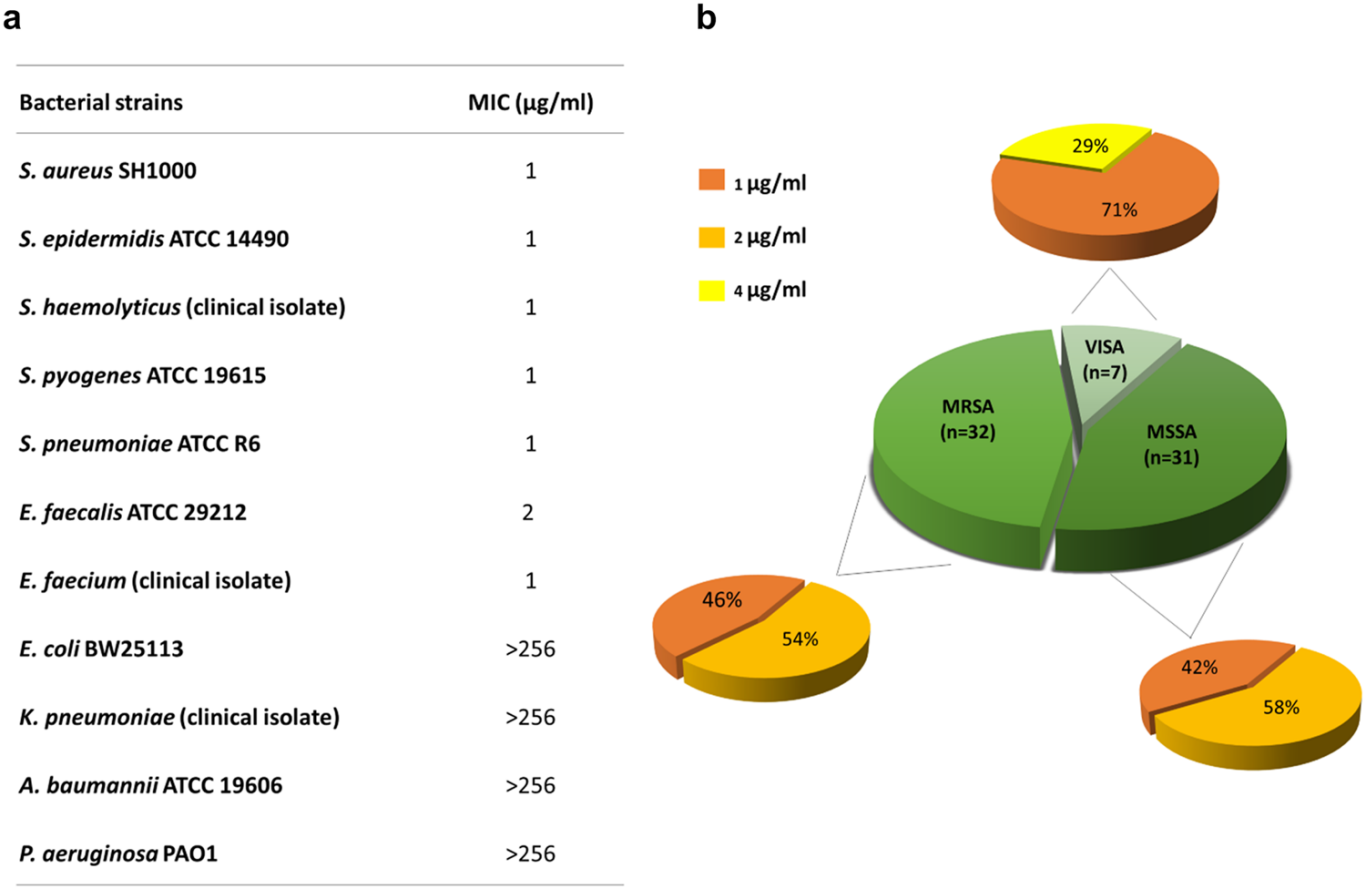
*In vitro* antibacterial activity of γ-ACT. (**a**) MICs (µg/ml) of **γ**-ACT against a panel of Gram-positive and Gram-negative bacteria. (**b**) MICs (µg/ml) for **γ**-ACT against a collection (*n* = 70) of clinical isolates of *S. aureus* comprising strains that are methicillin sensitive (MSSA), methicillin resistant (MRSA), and vancomycin intermediate (VISA). The central pie chart shows a breakdown of these different strain types in the collection, whilst the outer charts indicate the proportion of isolates susceptible to different concentrations of **γ**-ACT.

**γ**-ACT exhibited potent antibacterial activity against Gram-positive pathogens, displaying MIC values of 1–2 µg/ml against a cross-section of staphylococci, streptococci and enterococci (Fig. 2a). The antistaphylococcal activity of γ-ACT was further evaluated against a panel of 70 clinical isolates of *S. aureus* that included methicillin resistant *S. aureus* (MRSA) and vancomycin intermediate *S. aureus* (VISA) (Fig. 2b); the MIC_90_ of **γ**-ACT against these strains was 2 µg/ml (range of 1–4 µg/ml). Thus, the antistaphylococcal potency of **γ**-ACT is >10-fold greater than previously reported for compounds of this class^12^.

**γ**-ACT did not demonstrate useful antibacterial activity against Gram-negative pathogens, displaying MICs of >256 µg/ml against representative Enterobacteriaceae and non-fermentative bacilli (Fig. 2). Antibiotics that lack activity against Gram-negative bacteria usually do so because they are unable to traverse the outer membrane (OM) and/or are subject to efflux^15^. To examine whether one or both of these phenomena account for the failure of **γ**-ACT to inhibit the growth of *E. coli*, **γ**-ACT susceptibility determinations were conducted against *E. coli* BW25113 in the presence of an OM-permeabilising agent (polymyxin B nonapeptide: PMBN)^16^, and against derivatives of BW25113 deleted for components of the major efflux transporter, AcrAB-TolC. Whilst no change in the antibacterial activity of γ-ACT was observed against strains independently deleted for *acrA* or *tolC* (MICs of >256 µg/ml), the **γ**-ACT MIC dropped to 16 µg/ml against BW25113 in the presence of PMBN. This result implies that the limited activity of **γ**-ACT against Gram-negative bacteria like *E. coli* is, at least in part, a consequence of limited ingress across the OM, and establishes that the target of **γ**-ACT is present in Gram-negative bacteria.

**Figure 3:**
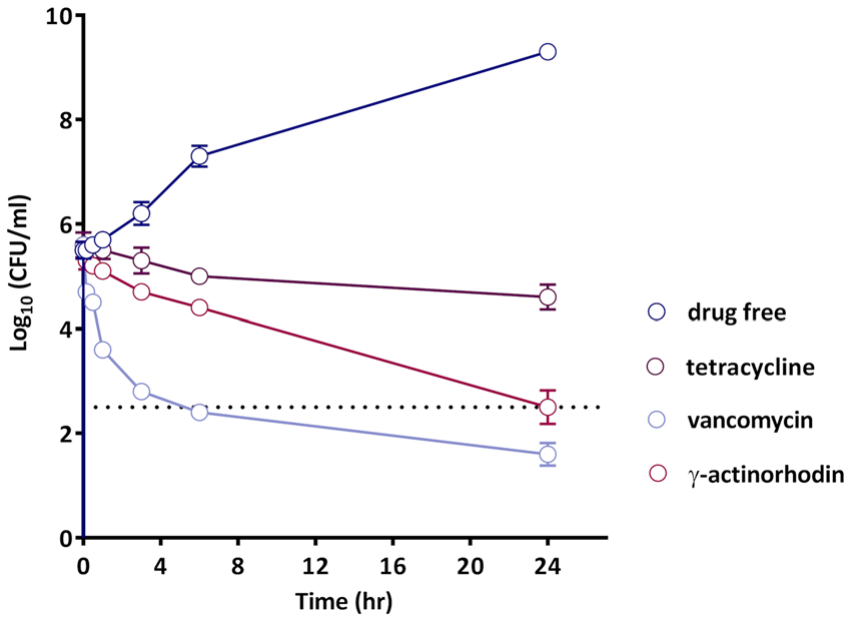
Time-dependent killing of exponential phase cultures of *S. aureus* SH1000 by γ-ACT and comparator agents at 4XMIC. The dotted line corresponds to a 3 log_10_ reduction in CFU/ml relative to cell density at time 0, and represents the threshold at which an antibiotic is considered to exhibit bactericidal activity. Values shown are the means of at least three independent experiments, with error bars representing standard deviations from the mean.

In time-kill experiments, **γ**-ACT was bactericidal at 4X MIC against rapidly growing cultures of *S. aureus* SH1000, mediating a reduction of ~3 log_10_ CFU/ml in viable cell numbers within an exponential phase population over 24 hours (Fig. 3). However, in common with most antibacterial drugs that exert a cidal action upon rapidly growing bacteria, **γ**-ACT showed negligible killing of non-growing (stationary phase) bacteria (*data not shown*).

### γ-ACT exhibits selective toxicity against bacteria

**Figure 4:**
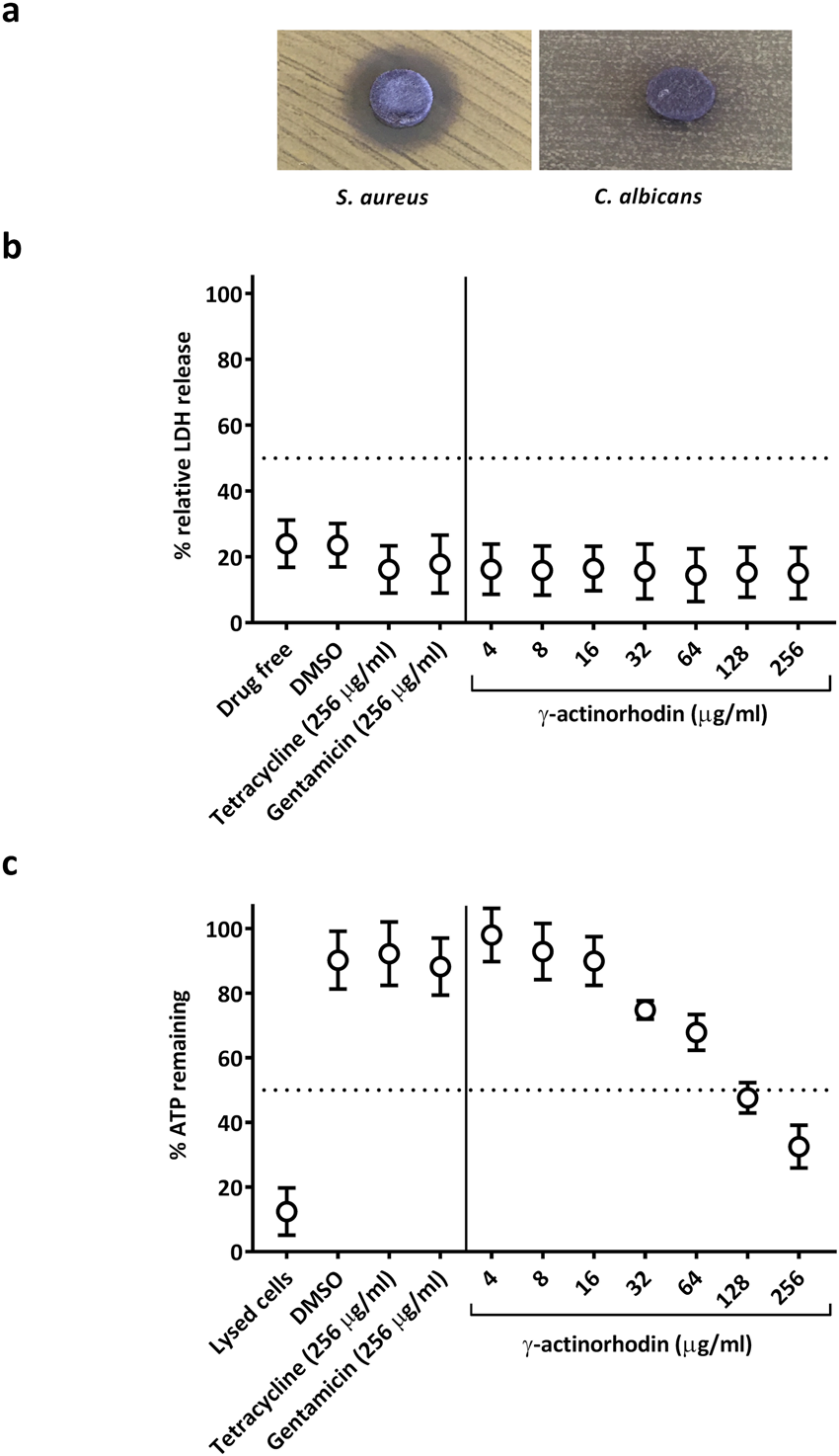
Prokaryotic selectivity and cytotoxicity of γ-ACT. γ-ACT inhibits the growth of the bacterium, *S. aureus*, but not that of the eukaryotic microorganism, *C. albicans* (**a**). The *in vitro* effect of γ-ACT and comparator agents on membrane integrity and viability of human kidney cells as determined by measuring release of lactate dehydrogenase (**b**) and cellular ATP levels (**c**), respectively. Values shown are the means of at least three independent experiments, with error bars representing standard deviations from the mean.

To provide a preliminary indication of prokaryotic selectivity, the activity of **γ**-ACT was evaluated against the eukaryotic microorganism, *Candida albicans*. Disk diffusion experiments revealed that **γ**-ACT exerts little activity against this organism when compared with that observed against *S. aureus* (Fig. 4a), and in MIC determinations, no inhibition of *C. albicans* was observed even at the highest concentration tested (256 μg/ml); these results imply that **γ**-ACT exhibits selectivity of action. Subsequently, we examined whether **γ**-ACT exerts any cytotoxic effects on mammalian cells by evaluating membrane integrity (monitored via release of lactate dehydrogenase) and viability (determined by measuring intracellular ATP levels) of human kidney-2 (HK-2) cells challenged with **γ**-ACT. **γ**-ACT exhibited no effect on HK-2 membrane integrity, even at the highest concentration tested (EC_50_ of >256 µg/ml) (Fig. 4b). However, a reduction in HK-2 viability was detected at higher concentrations of **γ**-ACT (EC_50_ of ~128 µg/ml) (Fig. 4c). Thus, whilst **γ**-ACT exhibited some cytotoxicity, this occurred at concentrations >50-fold higher than those required to achieve complete inhibition of bacterial growth, indicating that **γ**-ACT does indeed exhibit selective toxicity against bacteria.

### The mode of action (MOA) of γ-ACT involves collapse of the proton motive force and the generation of reactive oxygen species (ROS)

**Figure 5:**
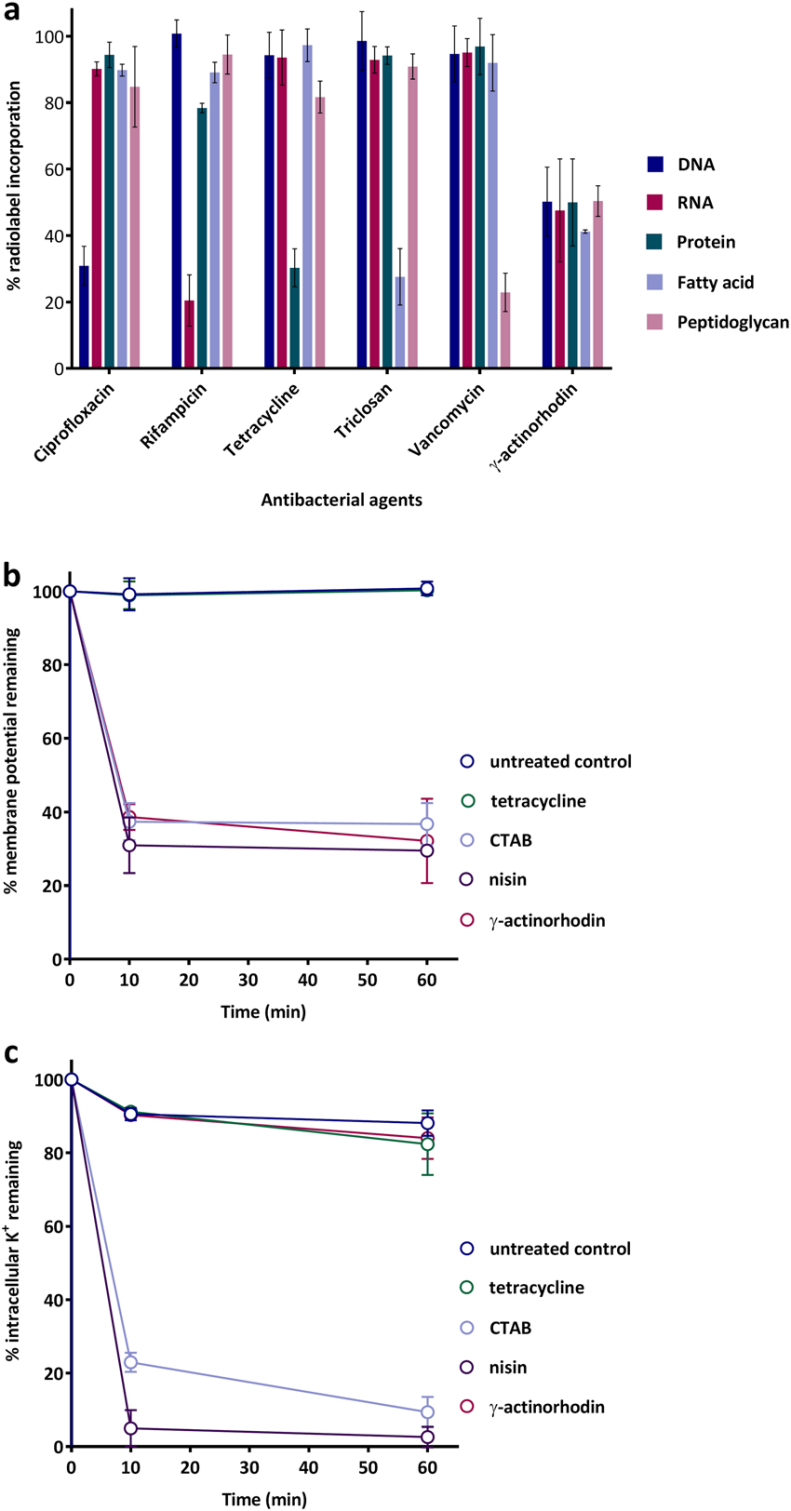
Antibacterial mode of action studies with γ-ACT. (**a**) Impact of a 10 minute challenge with γ-ACT and comparator agents at 4XMIC on macromolecular synthesis in *S. aureus* SH1000. (**b** and **c**) Effect of γ-ACT and comparator agents at 4XMIC on the function and integrity of the cytoplasmic membrane of *S. aureus* SH1000, as measured respectively in (**b**) with the PMF-responsive probe, DiSC_3_(5), and in (**c**) by monitoring leakage of K^+^ ions from cells. Values shown are the means of at least three independent experiments, with error bars representing standard deviations from the mean.

Most antibiotics in clinical use exert their antibacterial effects by inhibiting the biosynthesis of specific cellular macromolecules. To assess the effects of **γ**-ACT on macromolecular biosynthesis, we monitored incorporation of radiolabelled precursors into DNA, RNA, protein, fatty acid, and peptidoglycan, in *S. aureus* SH1000. At 4X MIC, **γ**-ACT caused substantial and comparable inhibition of all five biosynthetic pathways in 10 minutes (Fig. 5a). Non-specific inhibition of macromolecular biosynthesis is a characteristic of compounds that mediate their antibacterial effects through action on the cytoplasmic membrane^17,18^. We therefore sought to examine more directly whether **γ**-ACT exerts effects on the membrane of *S. aureus* SH1000. In experiments employing the fluorescent probe molecule, DiSC_3_ (5), **γ**-ACT at 4X MIC was found to cause substantial dissipation of the proton motive force (PMF) in 10 minutes, an effect comparable to that observed for the known membrane-active antibacterial agents, nisin and CTAB (Fig. 5b). In contrast to these control agents, however, **γ**-ACT did not cause physical perturbation of the membrane sufficient to permit leakage of ions (K^+^) from the cell (Fig. 5c).

ACTs are redox-active agents that have recently been reported to catalyse oxidation reactions *in vitro*, an observation that led those who reported it to propose that the antibacterial properties of this class might result from production of toxic levels of hydrogen peroxide^7^. In potential support of this idea, these investigators described modest restoration of staphylococcal growth in the presence of ACT when cultures were supplemented with catalase^7^. We took an orthogonal approach to explore a potential role for reactive oxygen species (ROS) in the antibacterial MOA of **γ**-ACT, examining whether strains of *S. aureus* defective in key components of the ROS protection response exhibit greater susceptibility to the inhibitory action of the antibiotic (Table 1). No change in the MIC of **γ**-ACT was observed against a derivative of *S. aureus* SH1000 (KS100) lacking the major catalase enzyme, KatA, or against a strain (KC043) completely devoid of catalase activity as a consequence of lacking both KatA and the alkyl hydroperoxide reductase^19^. However, we observed a substantial (8-fold) increase in susceptibility to **γ**-ACT for a strain of SH1000 lacking the major superoxide dismutase (SOD) enzyme, SodA (Table 1), and a further 2-fold increase in **γ**-ACT susceptibility for a strain concurrently lacking the other staphylococcal SOD, SodM (Table 1). By contrast, none of these strains exhibited a significant (>2-fold) increase in susceptibility to several comparator antibacterial agents, including those whose MOA involves action on the membrane (Table 1). Our results imply that superoxide, but not hydrogen peroxide, plays an important role in mediating the antibacterial effect of **γ**-ACT.

**Table 1:** Antibacterial activity of γ-ACT and comparator agents against *S. aureus* SH1000 and derivatives lacking enzymes important in the detoxification of ROS.

	Minimum inhibitory concentration (µg/ml)					
	γ-ACT	DAP	CTAB	VAN	CIP	RIF
SH1000	1	1	1	1	0.25	0.016
KS100	1	1	1	1	0.12	0.016
KC043	1	1	1	1	0.25	0.016
MHKA	0.12	1	1	1	0.12	0.016
MHKM	1	1	1	1	0.12	0.016
MHKAM	0.06	0.5	1	1	0.12	0.008

γ-ACT: γ-actinorhodin, DAP: daptomycin, CTAB: cetyltrimethylammonium bromide, VAN: vancomycin, CIP: ciprofloxacin, RIF: rifampicin.

### Inability to select resistance to γ-ACT *in vitro*

**Figure 6:**
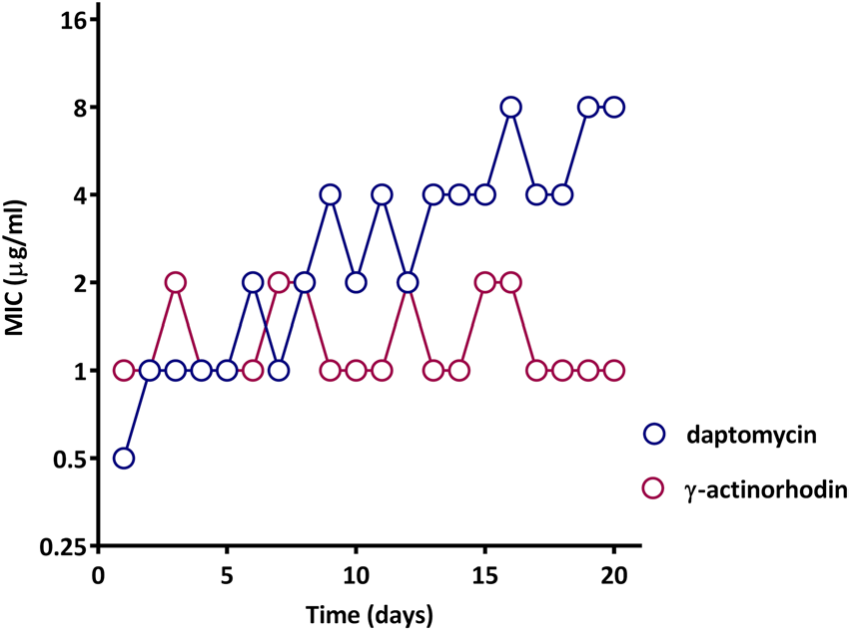
Resistance studies with γ-ACT. *S. aureus* SH1000 was serially-passaged in the presence of γ-ACT or the comparator compound (daptomycin) for 20 days. The data shown are representative of 3 independent experiments.

To evaluate the propensity for **γ**-ACT to select resistance, saturated cultures of *S. aureus* SH1000 were plated onto agar containing **γ**-ACT at multiples (4X-128X) of the (broth microdilution) MIC. Confluent growth was observed on agar containing up to 32X MIC **γ**-ACT, an observation that was subsequently explained by the finding that the antibacterial activity of **γ**-ACT becomes attenuated upon incorporation into agar (established by susceptibility testing using agar dilution; *data not shown*). A small number of colonies appeared on agar containing 64–128X MIC of **γ**-ACT after 48 hours’ incubation; however, these colonies were found to retain full susceptibility to **γ**-ACT in MIC determinations. We subsequently examined whether resistance to **γ**-ACT could be selected upon extended subculture in the presence of the compound. Serial passage of *S. aureus* SH1000 with **γ**-ACT was performed using the extended gradient MIC method^20^ that selects for resistance at both sub- and supra-MIC antibiotic concentrations in the same experiment, and may be considered to offer ‘optimal’ selection to favour the emergence of resistance. No reduction in **γ**-ACT susceptibility was observed in SH1000 over 20 days’ passage in the presence of the compound (Fig. 6). By contrast, over the same time period we recorded a substantial reduction (16-fold) in susceptibility to the comparator antibiotic, daptomycin, a drug that is generally considered to have low resistance potential.

### Safety and antibacterial efficacy *in vivo*

**Figure 7:**
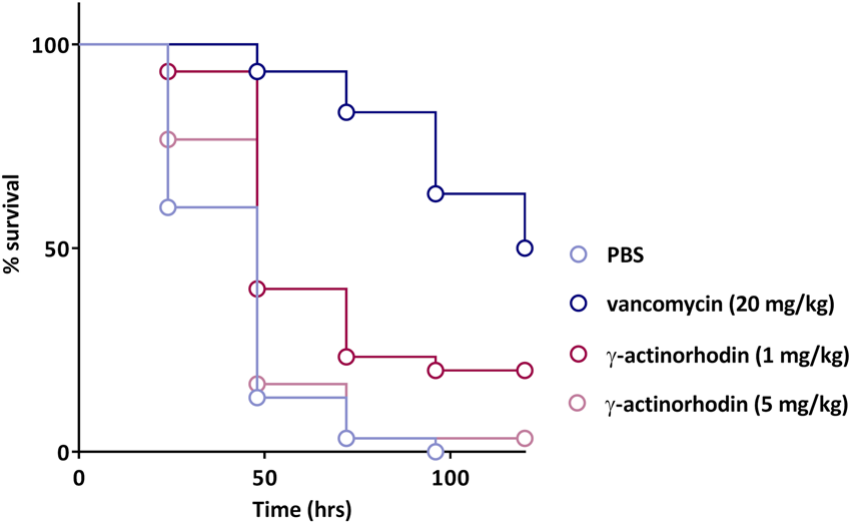
Effect of γ-ACT on survival of *G. mellonella* infected with MRSA. Groups of 30 larvae were challenged with *S. aureus* JE2 (1 × 10^7^ CFU) and then treated with γ-ACT at concentrations of 1 or 5 mg/kg or with vancomycin at 20 mg/kg 30 mins post infection. The number of live *vs* dead larvae was determined every 24 h post-infection up to 120 h.

*In vivo* studies employed larvae of the greater waxmoth (*Galleria mellonella*). In initial experiments, we established that **γ**-ACT does not exert overt toxic effects *in vivo*, with 80% (8/10) *G. mellonella* larva surviving for at least 48 hours’ post-administration of **γ**-ACT at 50 mg/kg (the highest concentration tested), and 100% (10/10) surviving following administration of **γ**-ACT at 20 mg/kg. Subsequently, we established infection in *G. mellonella* using the virulent MRSA USA300 strain, JE2, and used this model to evaluate the *in vivo* efficacy of **γ**-ACT. Following infection at an inoculum of 10^7^ CFU, all of the insects died 96 hours’ post infection (pi) in the absence of antibiotic treatment. Analysis of survival curves (Fig. 7) using the Log-rank test for trend showed a global statistical significance (*P* < 0.0001). Subsequent pairwise analysis indicated that administration of vancomycin (20 mg/kg) 30 minutes pi significantly enhanced *G. mellonella* survival (*P* < 0.0001) when compared to the no-treatment control, with 50% of the insects alive at 120 hours pi. We observed modest but significant (*P* = 0.0003) protection following administration of **γ**-ACT compared to no-treatment control at low concentrations 30 minutes pi; **γ**-ACT at 1 mg/kg rescued 20% of the insects as judged 120 hours’ pi (Fig. 7). At a higher concentration of **γ**-ACT (5 mg/kg), the protective effect was not significant (*P* = 0.2365), with survival at 120 hours pi dropping to ~3% (Fig. 7). At higher concentrations still (20 and 50 mg/kg), administration of **γ**-ACT to infected *G. mellonella* not only failed to yield a protective effect, but actually appeared to act in concert with the infection to kill the insects; all infected insects treated with **γ**-ACT at these concentrations were dead by 72 hours (i.e. before those infected insects not treated with **γ**-ACT) (*data not shown*). Thus, whilst **γ**-ACT does display modest antibacterial efficacy *in vivo* when administered at low concentration, this effect is lost at higher concentrations.

## Discussion

We have described here the antibacterial properties of **γ**-ACT, and thereby provided the first evaluation of a member of the ACT class as a potential antibacterial drug candidate.

**γ**-ACT exhibits potent antibacterial activity that encompasses two of the ESKAPE pathogens (*S. aureus* and *E. faecium*), with MICs against members of these species comparable to those seen for antibacterial drugs in current clinical use^18^. As indicated above, the antibacterial potency of **γ**-ACT is substantially greater than that previously published for ACT; in contrast to the MIC of 1 µg/ml we recorded against our laboratory strain of *S. aureus* in a broth microdilution experiment, the original study that reported on the antibacterial activity of this class estimated an antistaphylococcal MIC of 25–30 µg/ml by agar diffusion^12^. Whilst there are several explanations that could account for this apparent discrepancy, we consider that it is likely predominantly due to the phenomenon we observed for **γ**-ACT, whereby the antibacterial activity becomes attenuated in agar. The weak activity originally reported for ACT^12^ probably explains why this class has not been further evaluated in respect of its therapeutic potential over the last 40 years, and it is intriguing to consider that other potentially interesting/useful antibiotic classes are languishing ‘obscured’ in the scientific literature owing to similar points of technicality.

The antibacterial mechanism of **γ**-ACT involves rapid dissipation of the PMF, prompting comprehensive shutdown of macromolecular biosynthesis, and ultimately, cell death. Several other antibacterial agents in use as drugs (daptomycin, telavancin), antiseptics (CTAB) or food preservatives (nisin) also possess an MOA that involves disruption of membrane energetics^21–23^. However, in contrast to **γ**-ACT, these agents additionally cause gross physical perturbation of the membrane, leading to detectable leakage of intracellular contents. Thus, the action of γ-ACT on the bacterial membrane is apparently distinct from that of other membrane-perturbing agents in use. Nor is the antibacterial action of **γ**-ACT restricted exclusively to effects at the membrane. The dramatic sensitization to **γ**-ACT observed in *S. aureus* strains lacking superoxide dismutases, enzymes that constitute the major cellular defence against superoxide, strongly implicates this radical in the MOA of **γ**-ACT. Sensitization to **γ**-ACT was predominantly associated with loss of SodA, an enzyme that has been linked specifically with protection against internally-generated oxidative stress in *S. aureus* ^24^, implying that **γ**-ACT prompts the generation of endogenous, rather than exogenous, superoxide. It seems probable that these two antibacterial mechanisms observed for **γ**-ACT (collapse of the PMF and the production of ROS) share a common root cause; both effects could be explained by **γ**-ACT-mediated interference with correct functioning of the electron transport chain, a process that ordinarily not only acts to generate the PMF, but which also represents the primary source of superoxide in the bacterial cell^25^. Thus, we speculate that **γ**-ACT mediates oxidative damage to one or more components of the electron transport chain, which in turn acts both to compromise the bacterium’s ability to maintain the PMF and yields a source of free electrons to drive the generation of superoxide.

Compounds that exert their antibacterial effects through action at the bacterial membrane – including those that act to interfere with membrane energetics - are a frequent occurrence in antibacterial drug discovery programmes^18,26^. Such agents are usually considered nuisance compounds in this context, since their action on biological membranes is almost invariably non-specific, and as a consequence, they lack prokaryotic selectivity^2,18^. **γ**-ACT, whilst exhibiting an effect on membrane energetics comparable to that mediated by such compounds, does however exhibit prokaryotic selectivity; the concentration of this compound required to produce a significant cytotoxic effect was at least 50X that required to completely inhibit bacterial growth. Future elucidation of the precise mechanism by which **γ**-ACT achieves this selectivity could potentially inform the generation of other membrane-active antibacterial drug candidates.

In view of the selective nature of **γ**-ACT, we evaluated the therapeutic potential of this compound in an invertebrate model of staphylococcal infection. **γ**-ACT at 1 mg/kg demonstrated a modest but significant protective effect *in vivo*, rescuing 20% of infected insects – a figure just less than half that seen (50%) for the established antibacterial drug, vancomycin. Whilst **γ**-ACT therefore appears to possess some therapeutic potential, further studies will be required to understand why the compound not only fails to improve survival of infected *G. mellonella* when administered at higher concentrations, but beyond a certain concentration, actually acts to reduce it. Potentially, whilst not overtly cytotoxic at the concentrations administered, **γ**-ACT may compromise the immune response in *G. mellonella*, which would in turn act to facilitate the progress of the infection. We note that several other compounds have been reported to increase the susceptibility of *G. mellonella* to infection, an effect that results from inhibition of haemocyte function^27,28^.

Whether such a phenomenon is also evident for **γ**-ACT in higher organisms remains to be established. However, even if that does transpire to be the case, that need not preclude **γ**-ACT from further consideration as a candidate topical agent to prevent and treat staphylococcal disease, or as a chemical starting point for the generation of derivatives with properties better suited for systemic application. This is particularly the case since **γ**-ACT otherwise possesses many of the requisite properties of a useful antibacterial drug^18^; in addition to potent and selective bactericidal activity, an MOA distinct from that of other antibacterial drugs in clinical use, and evidence for *in vivo* efficacy, **γ**-ACT exhibits extremely low resistance potential *in vitro*. We consider our findings to underscore the utility of revisiting unexploited antibiotics as a potential source of novel antibacterial drug candidates, and believe that a comprehensive re-evaluation of such compounds is now warranted.

## Materials and Methods

### Microorganisms and growth media

Laboratory strains of bacteria and yeast used in this study are listed in Table 2. Clinical isolates of *S. aureus* were part of a culture collection belonging to the Antimicrobial Research Centre, University of Leeds, UK. The ACT over-producer strain, *Streptomyces coelicolor* L646 (kindly provided by K. McDowall, University of Leeds), was used as the source of **γ**-ACT, and was propagated on ISP medium 2 (Difco) containing 50 µg/ml apramycin at 30 °C. Unless otherwise stated, experiments to evaluate the antibacterial properties of **γ**-ACT employed *Staphylococcus aureus* SH1000. Bacteria (with the exception of *S. coelicolor*) were routinely cultured in Mueller-Hinton broth (MHB) and on Mueller-Hinton agar (MHA) (Oxoid Ltd, Cambridge, UK) for 24 h at 37 °C, whilst *Candida albicans* was grown in lysogeny broth (LB) and on LB agar (Oxoid) for 48 hours at 35 °C.

**Table 2:** Microorganisms used in this study.

Strain	Description	References
*Streptomyces coelicolor* L646	*S. coelicolor* M145 containing an integrated plasmid overexpressing wild-type *atrA*, which leads to hyper-production of actinorhodins	33
*Staphylococcus aureus* SH1000	Standard laboratory strain - derivative of strain 8325-4, with a functional *rsbU* gene reinstated	34,35
*S. aureus* JE2	Methicillin-resistant *S. aureus* USA300 strain	36
*S. aureus* KS100	SH1000 deficient in catalase (*katA*::Tn*917*)	19
*S. aureus* KC043	SH1000 deficient in alkyl hydroperoxide reductase and catalase (*ahpC*::*tet*, *katA*::Tn*917*)	19
*S. aureus* MHKA	SH1000 deficient in superoxide dismutase A (*sodA*::Tn*917*)	37
*S. aureus* MHKM	SH1000 deficient in superoxide dismutase M (*sodM*:: *tet*)	37
*S. aureus* MHKAM	SH1000 deficient in superoxide dismutases A and M (*sodA*::Tn*917*, *sodM*::*tet*)	37
*Escherichia coli* BW25113	Derivative of *E. coli* K12 strain BD792 and parent strain of the Keio collection	38,39
*E. coli* BW25113*ΔacrAB*	BW25113 deleted for AcrAB	38
*E. coli* BW25113*ΔtolC*	BW25113 deleted for TolC	38
*Candida albicans* CA-6	Clinical isolate	40

### Antibacterial agents and chemicals

Antibacterial agents were from Sigma-Aldrich (Poole, UK) with the following exceptions; ciprofloxacin (Alfa Aesar, Lancashire, UK), vancomycin (Cayman Chemical, Michigan, USA), daptomycin (Cubist Pharmaceuticals, Massachusetts, USA) and nisin (Duchefa Biochemie, Haarlem, Netherlands). Radiolabelled chemicals were from PerkinElmer (Waltham, Massachusetts, USA); unless otherwise stated, all other chemicals were from Sigma-Alrdich.

### Extraction, purification and chemical characterization of γ-ACT

*S. coelicolor* L646 was grown on ISP 2 agar at 30 °C for 6 days. The agar was manually fragmented and the pigment extracted twice with ethyl acetate at 30 °C for an hour with vigorous shaking. The resulting extract was dried *in vacuo*, dissolved in chloroform (60 mL), and washed with aqueous sodium carbonate solution (pH 10.9, 60 ml × 3). The combined blue aqueous layer was acidified with hydrochloric acid (6 M), yielding a crude red pigment that was recovered by filtration. γ-ACT was then triturated from methanol (red precipitate, 125 mg, 19%). R_*F*_ 0.29 (1% MeOH in dichloromethane). The identity and purity of γ-ACT were confirmed using mass spectrometry, 1-D NMR, 2-D NMR, infrared spectroscopy and analytical HPLC.

### Evaluation of antimicrobial activity

Susceptibility testing with **γ**-ACT against bacteria was performed by broth microdilution in MHB according to CLSI guidelines^29^. Antifungal activity was assessed in essentially the same way, though used LB in place of MHB, and minimum inhibitory concentrations (MICs) were instead read after 48 hours’ incubation at 35 °C. In both antibacterial and antifungal susceptibility tests the MIC was defined as the lowest concentration of antibiotic that completely inhibited visible growth. To assess the role of the outer membrane in restricting entry of **γ**-ACT into *E. coli*, polymyxin B nonapeptide (PMBN; Sigma-Aldrich) was added to MIC determinations at a final concentration of 4 µg/ml^16^.

Time-kill studies on exponential-phase populations of S*. aureus* SH1000 were performed using standard methodology^30^, utilizing ~5 × 10^5^ CFU/ml in MHB and antibiotics at 4X MIC. For time-kill studies on non-growing bacteria, stationary-phase cells were resuspended in spent medium at a density of ~5 × 10^5^ CFU/ml. Following antibiotic challenge, bacterial viability was monitored by plating cultures onto MHA, and enumerating colonies after incubation at 37 °C for 18–24 h.

### Cytotoxicity testing

Human Kidney 2 cells (ATCC CRL-2190) were obtained from the American Type Culture Collection and maintained in high-glucose Dulbecco’s Modified Eagle’s medium (DMEM) supplemented with 1% of penicillin-streptomycin combo solution and 10% fetal bovine serum (Sigma-Aldrich, Poole, UK). Cells were cultured at 37 °C in 5% CO_2_ saturated air, replacing the culture media every 48 hours. Cells were passaged upon reaching 80% confluency, and seeded in 96-well flat bottom plates at a density of 1.2 × 10^4^ cells/well in 200 μl of DMEM medium without serum. After a further 24 hours’ culturing, cells were exposed to the antibiotic under test for 6 hours and cytotoxicity assessed by measuring lactate dehydrogenase leakage with the CytoTox-ONE^TM^ Homogenous Membrane Integrity Assay (Promega, USA), and by monitoring intracellular ATP levels using the Adenosine 5′-triphosphate bioluminescent somatic cell assay kit (Sigma-Aldrich, UK).

### Mode of action studies

The effect of **γ**-ACT on macromolecular synthesis was determined in mid-exponential phase cultures (10^8^ CFU/mL in LB) of *S. aureus* SH1000 by monitoring incorporation of radiolabelled precursors into TCA-precipitable material following a 10 minute challenge with the compound at 4X MIC^17^. The radioactive precursors [methyl-^3^H] thymidine (70–95 Ci/mmol), [5, 6–^3^H] uridine (31–56 Ci/mmol), [3,4-^3^H(N)] glutamine (20–50 Ci/mmol), [1,2-^14^C] acetic acid (45-60 mCi/mmol), and ^14^C (U)-glycine (>80 mCi/mmol) were used to monitor the synthesis of DNA, RNA, protein, fatty acid and peptidoglycan, respectively, and were added to bacterial cultures 10 minutes prior to the addition of antibiotics. The effect of **γ**-ACT on the proton motive force was assessed using 3, 3’-diprophlthiadicarbocyanine iodide [DiSC_3_(5)] (Invitrogen, Paisley, UK)^23^, whilst physical damage to the membrane was evaluated by measuring leakage of potassium ions, as previously described^22^.

### Resistance studies

Initial attempts to select mutants exhibiting reduced susceptibility to **γ**-ACT involved plating saturated (>10^9^ CFU/ml) cultures of *S. aureus* SH1000 onto MHA containing (4X-128X) MIC of **γ**-ACT, and incubation for 48 hours at 37 °C. To select resistance by serial passage, *S. aureus* SH1000 cells were continuously exposed to **γ**-ACT for 20 days using the extended gradient MIC method^20^.

### Safety and efficacy studies in *Galleria mellonella*

*G. mellonella* (greater wax moth) larvae from Livefood UK Ltd (Rooks Bridge, Somerset, UK) were maintained on wood chips in the dark at 14 °C for up to 2 weeks. Bacterial infection and subsequent treatment of *G. mellonella* was performed on 30 larvae (3 groups of ten) per treatment using established methodology^31^, with antibiotic treatment of infection undertaken as described^32^. Briefly, 10 µl volumes containing 1 × 10^7^ CFU of MRSA strain JE2 were injected into the bottom left proleg of each individual larva. Antibiotics were subsequently administered in phosphate-buffered saline (PBS) by injection 30 minutes post-infection, with PBS administered alone as a no-treatment control to account for the increase in liquid volume within the *G. mellonella*. Antibiotic concentrations achieved in *G. mellonella* were estimated on an average larva weight of 300 mg. The number of larvae alive/dead was calculated every 24 hours up to 120 hours when the experiment was halted, and the resulting data analysed using Prism Software Version 6 (Graphpad, San Diego, CA, USA). Kaplan-Meier curves were plotted and a global statistical comparison carried out using the Log-Rank test for trend; individual treatments were compared with the no-treatment (PBS) control using the Mantel-Cox (Log-Rank) statistical test. The effectiveness of a particular treatment was considered significant if *P* < 0.05.

### Data availability

The majority of the data generated or analysed during this study are included in this published article and its Supplementary Information files; data not shown are available from the corresponding author upon request.

## Electronic supplementary material

Supplementary information
