# Supplementary material for: Revisiting unexploited antibiotics in search of new antibacterial drug candidates: the case of γ-actinorhodin
Source: Sci Rep. 2017 Dec 12;7:17419. doi: 10.1038/s41598-017-17232-1 (PMC5727142; doi:10.1038/s41598-017-17232-1)
Supplement: Supplementary file 1 — Supplementary information [file 41598_2017_17232_MOESM1_ESM.pdf]

Supplementary information for:

Revisiting unexploited antibiotics in search of new antibacterial drug candidates: the case of γ-actinorhodin

Nada M. Nass, Sannia Farooque, Charlotte Hind, Matthew E. Wand, Christopher P. Randall, J. Mark Sutton, Ryan F. Seipke, Christopher M. Rayner and Alex J. O'Neill

Identification and characterization of γ-actinorhodin

γ-actinorhodin was isolated as red precipitate (125 mg, 19% *w/w*). R_F 0.29 (1% MeOH in dichloromethane); δ_H (500 MHz, $CDCl_3$) 12.80 (1H, s, OH-9), 12.50 (1H, s, OH-6), 7.34 (1H, s, H-7), 5.33 (1H, d, *J* 3.0 Hz, H-4), 5.19 (1H, q, *J* 7.0 Hz, H-1), 4.74 (1H, m, H-3), 2.99 (1H, dd, *J* 17.8, 5.3 Hz, H-11), 2.73 (1H, d, *J* 17.8 Hz, H-11′), 1.59 (3H, d, 7.0 Hz, CH_3); δ_C (125 MHz, $CDCl_3$) 176.90 (C-5), 176.6 (C-10), 174.1 (C-12), 165.67 (C-6), 164.91 (C-9), 148.88 (C-10a), 138.04 (C-8), 133.68 (C-7), 133.35 (C-4a), 112.05 (C-9a), 111.72 (C-5a), 68.68 (C-4), 66.84 (C-1), 66.48 (C-3), 37.02 (C-11), 18.51 (CH_3); HRMS (ESI^-): m/z calculated for formula $C_{32}H_{21}O_{14}$ [M-H] 629.0933; found 629.0922; IR (ν_{max}, solid, cm^{-1}): 3502, 2919, 2850, 1790, 1709, 1613, 1575, 1377, 1260.

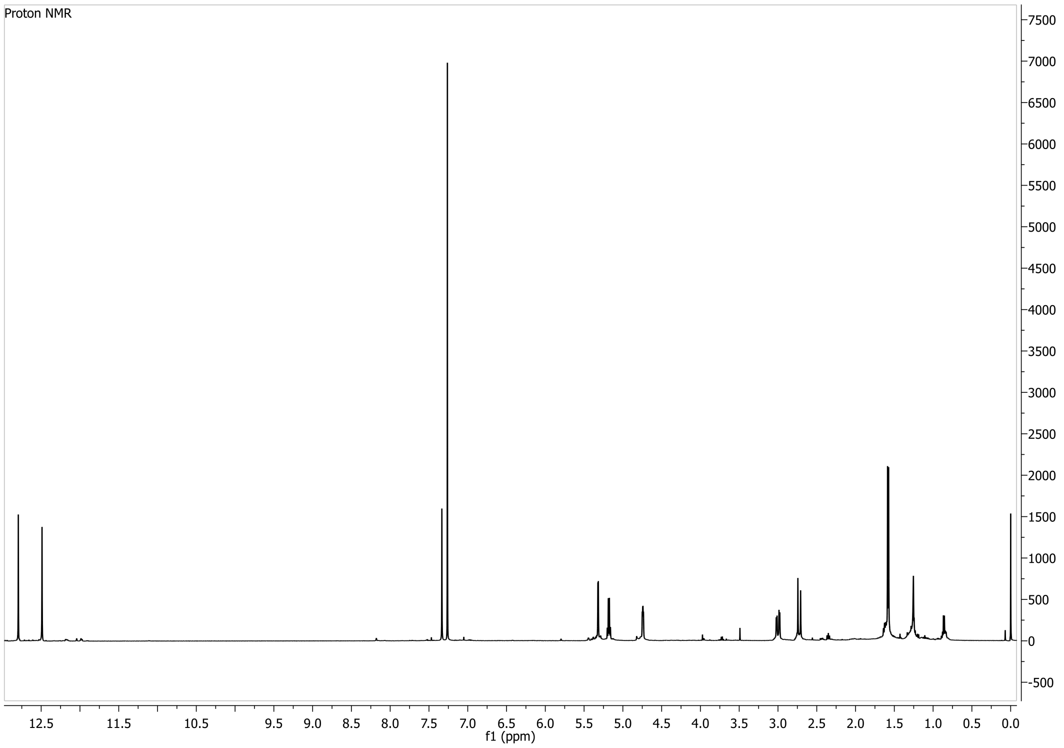

^1H NMR spectrum for γ-actinorhodin

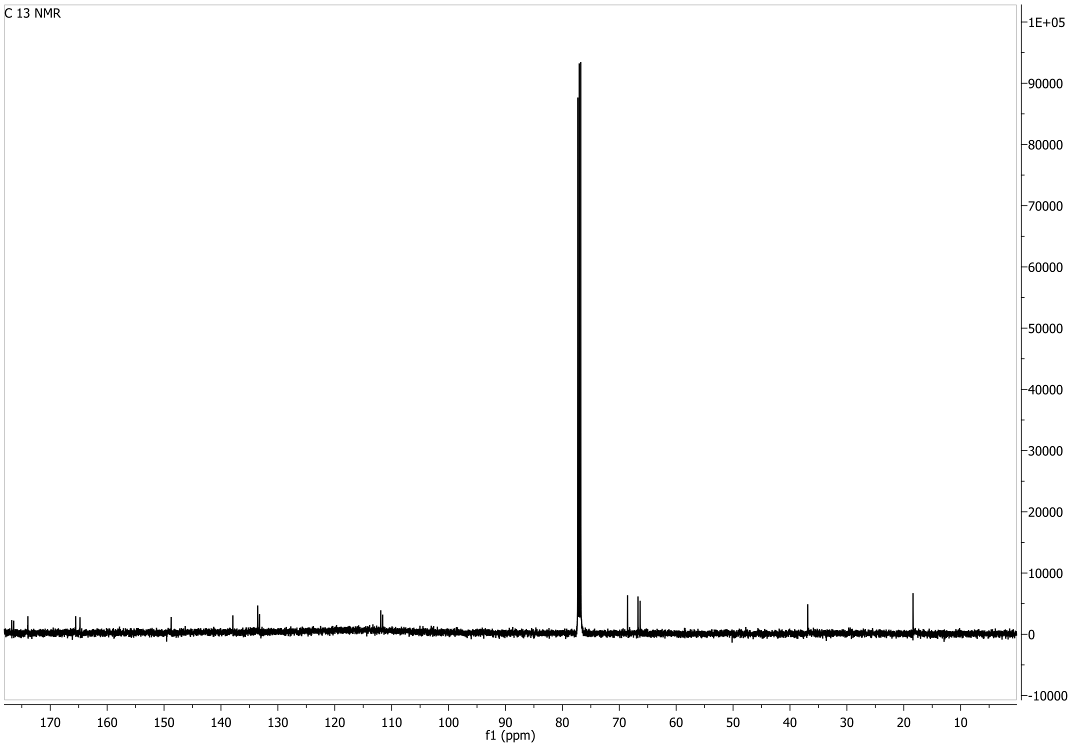

^{13}C spectrum for γ-actinorhodin

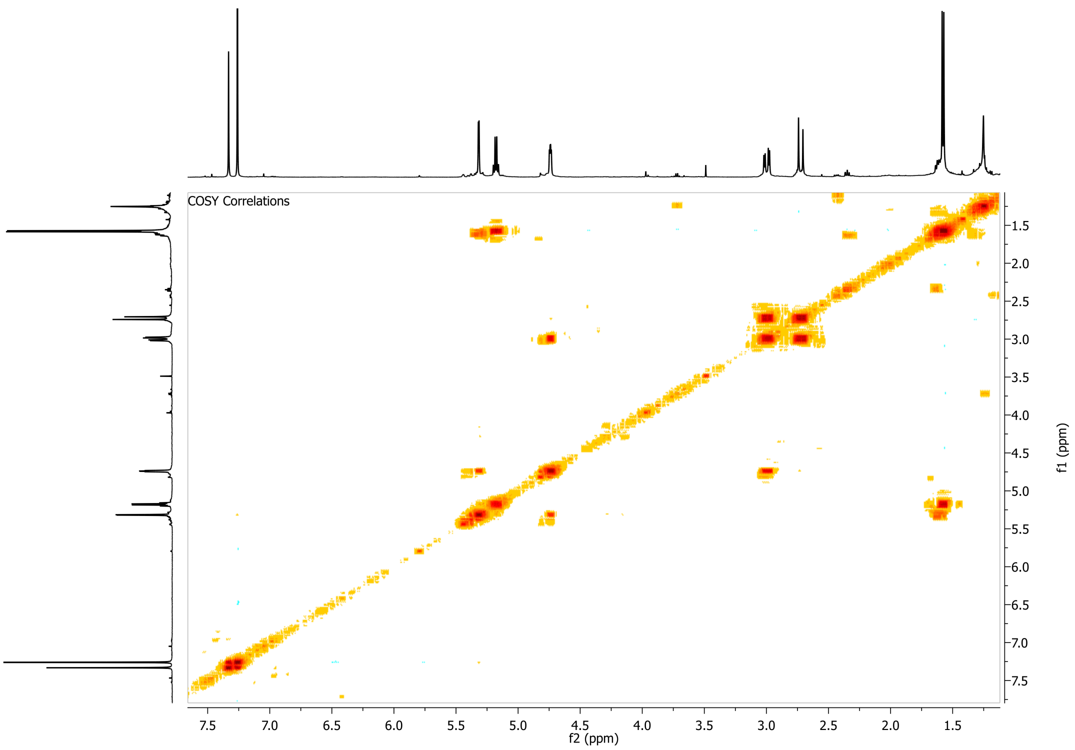

^1H-^1H COSY spectrum for γ-actinorhodin

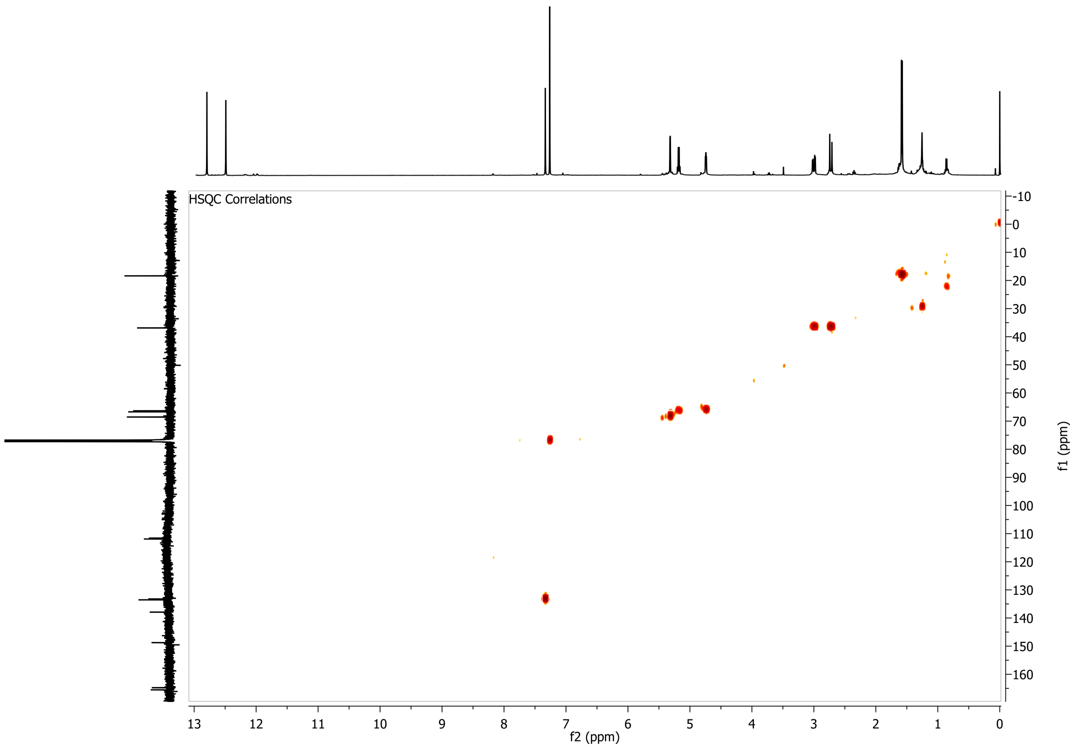

^{13}C-^{1}H HSQC spectrum for γ-actinorhodin

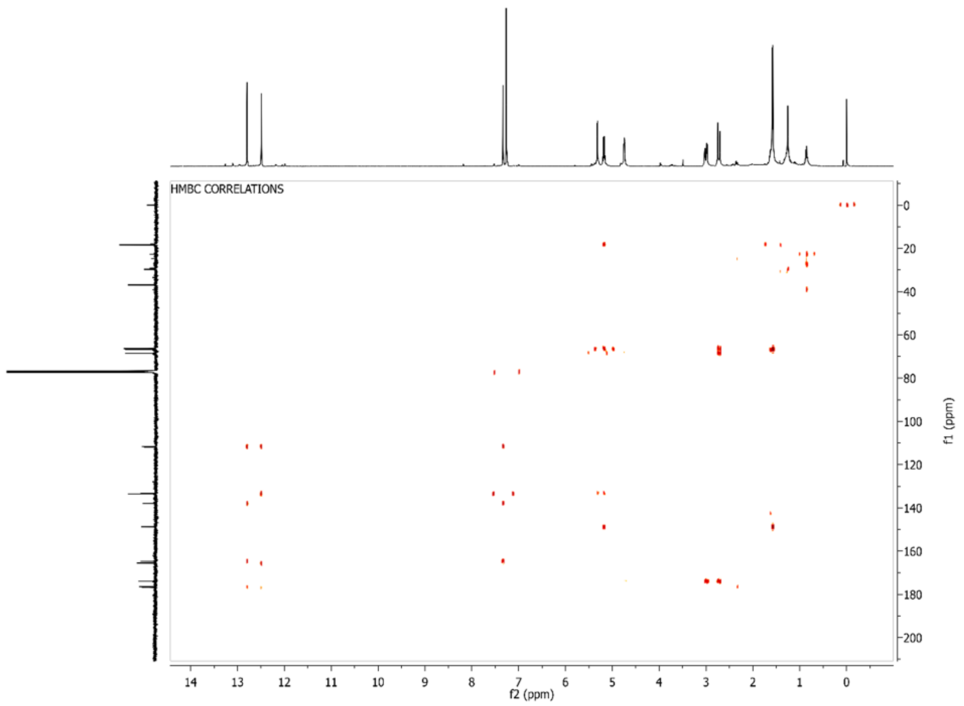

^{13}C-^{1}H HMBC spectrum for γ-actinorhodin

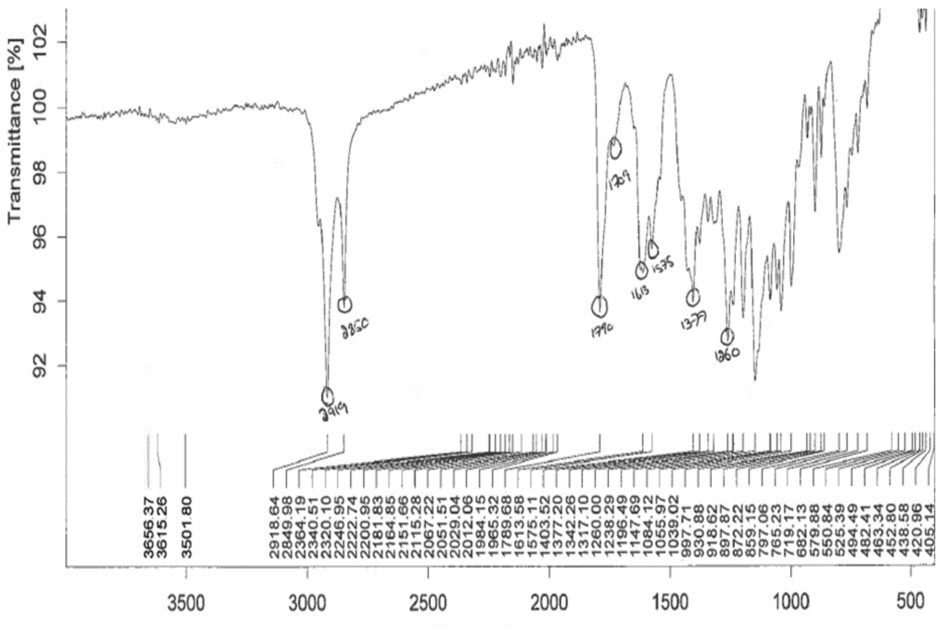

IR spectrum for γ-actinorhodin

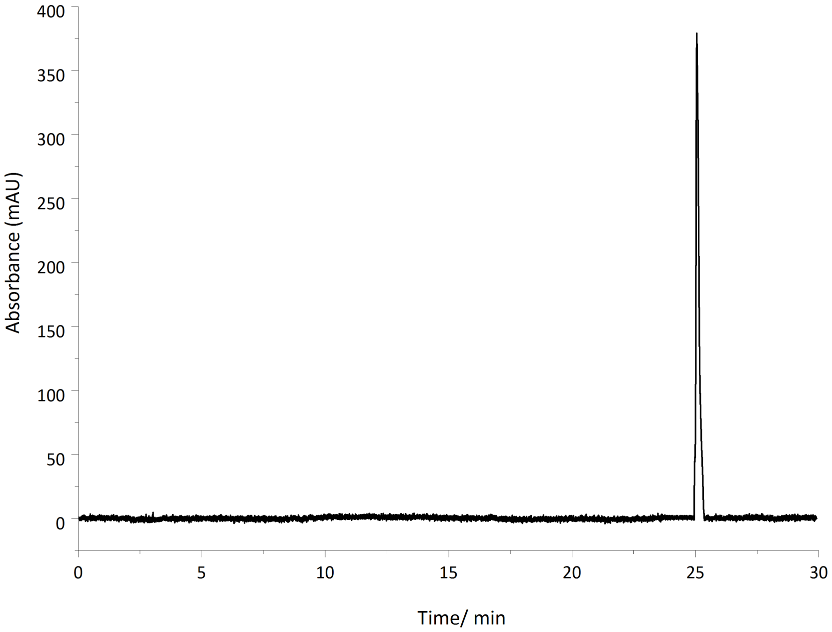

HPLC chromatogram recorded for γ-actinorhodin in acetone at 520 nm
